# A proteomic analysis shows the stimulation of light reactions and inhibition of the Calvin cycle in the skin chloroplasts of ripe red grape berries

**DOI:** 10.3389/fpls.2022.1014532

**Published:** 2022-10-25

**Authors:** António Teixeira, Henrique Noronha, Mónica Sebastiana, Ana Margarida Fortes, Hernâni Gerós

**Affiliations:** ^1^ Centre of Molecular and Environmental Biology (CBMA), Department of Biology, University of Minho, Braga, Portugal; ^2^ BioISI – Instituto de Biosistemas e Ciências Integrativas, Faculdade de Ciências, Universidade de Lisboa, Lisbon, Portugal

**Keywords:** grape berry skin, chloroplasts, proteomics, photosynthesis, *Vitis vinifera*

## Abstract

The role of photosynthesis in fruits still challenges scientists. This is especially true in the case of mature grape berries of red varieties lined by an anthocyanin-enriched exocarp (skin) almost impermeable to gases. Although chlorophylls are degraded and replaced by carotenoids in several fruits, available evidence suggests that they may persist in red grapes at maturity. In the present study, chloroplasts were isolated from the skin of red grape berries (cv. Vinhão) to measure chlorophyll levels and the organelle proteome. The results showed that chloroplasts (and chlorophylls) are maintained in ripe berries masked by anthocyanin accumulation and that the proteome of chloroplasts from green and mature berries is distinct. Several proteins of the light reactions significantly accumulated in chloroplasts at the mature stage including those of light-harvesting complexes of photosystems I (PSI) and II (PSII), redox chain, and ATP synthase, while chloroplasts at the green stage accumulated more proteins involved in the Calvin cycle and the biosynthesis of amino acids, including precursors of secondary metabolism. Taken together, results suggest that although chloroplasts are more involved in biosynthetic reactions in green berries, at the mature stage, they may provide ATP for cell maintenance and metabolism or even O2 to feed the respiratory demand of inner tissues.

## Introduction

In higher plants, whether photosynthesis can occur in sink organs is still a matter of debate. Fruit photosynthesis has been studied in coffee, peas, soybeans, avocados, oranges, apples, and grape berries (Quebedeaux and Chollet, 1975; Atkins et al., 1977; Blanke and Lenz, 1989; Lopez et al., 2000; Aschan and Pfanz, 2003; Breia et al., 2013; Garrido et al., 2018), where it may contribute additional organic carbon to plant growth (Aschan and Pfanz, 2003), O_2_ for respiration, and secondary metabolites biosynthesis (Rolletschek et al., 2003; Breia et al., 2013; Garrido et al., 2018). It could even refix CO_2_ produced during mitochondrial respiration (Blanke and Lenz, 1989). Nonetheless, the role of photosynthesis in fruits is far from being completely understood.

Grape berries are composed of different tissues and cell layers with distinct anatomical characteristics and biochemical profiles that play distinct roles during development and ripening. The exocarp (or skin) is formed by an epidermis covered with an outer waxy cuticle and a hypodermis that has up to 17 layers of collenchymatous cells (Considine and Knox, 1979; Hardie et al., 1996; Breia et al., 2013). Early observations suggested that ripe berries have no stomata (Pratt, 1971), but recent scanning electron microscopy showed very few yet functional stomata in young berries and wax-filled stomata in older berries (Rogiers et al., 2004). Nonetheless, depending on the cultivar, the abundance of stomata in young berries may be 100-fold less than in the abaxial epidermis of a typical leaf (Blanke and Leyhe, 1988; Blanke and Lenz, 1989; Rogiers et al., 2011). In young fruits, stomata are as sensitive in leaves and regulate the rate of CO_2_ exchange to a certain extent, while in the ripening fruit, the cuticular component and mostly unregulated lenticels dominate the diffusive resistance to CO_2_ (Aschan and Pfanz, 2003). The gradual disappearance of stomata and/or the development of an impermeable waxy cuticle during development results in an internal environment characterized by high CO_2_ and low O_2_ levels (Blanke and Leyhe, 1987; Blanke and Leyhe, 1988).

In contrast to the planar morphology of leaves, the large volumetry of fruits, especially fleshy fruits, imposes physical constraints on light penetration into the inner tissues and restricts the photic zone to the outermost layers (Breia et al., 2013). Light transmission through the skin of *Vitis* berries was reported to reach 47% of the incident photon flux density and only up to 2% reaches the innermost regions (Aschan and Pfanz, 2003). A clear tissue-specific distribution pattern of photosynthetic competence was observed in the white grape berries from cv. Alvarinho (Breia et al., 2013). The exocarp revealed the highest photosynthetic capacity and the lowest susceptibility to photoinhibition. Meanwhile, low fluorescence signals and photochemical competence were found in the mesocarp. Recent studies of the same variety have shown that the photosynthetic activity of the exocarp was responsive to low and high light microclimate intensity differences in the canopy (Garrido et al., 2019). Because chlorophyll pigments are kept during the transition to the mature stage, although in lower amounts than in green berries (Giovanelli and Brenna, 2007; Kamffer et al., 2010), the accumulation of anthocyanins accounts for the change in color in red varieties. This is the case for Merlot, where the amount of photosynthetic chlorophyll pigments decreases from 19 to 10 μg/berry fresh weight (FW). Contrarily, chlorophylls are replaced by carotenoids during the ripening of tomatoes (Bean et al., 1963; Stiles, 1982; Blanke and Lenz, 1989), thus, fruit metabolism changes from partially photosynthetic (12 to 39 μg *chl* g FW^–1^ chlorophyll) at the green stage to truly heterotrophic at the mature stage (Lytovchenko et al., 2011). This transition appears to be coupled with a decline in the expression and enzymatic activities associated with carbon assimilation (Lytovchenko et al., 2011).

Hints at the grape berries’ photosynthetic competence during development and ripening have been provided at the gene and protein levels in whole berries, berry pulp, and skin. In general, transcripts encoding proteins associated with photosynthesis-related functions are strongly expressed during phase I of berry development (Terrier et al., 2005; Waters et al., 2005; Deluc et al., 2007; Grimplet et al., 2007; Dal Santo et al., 2013; Ghan et al., 2017), while genes encoding Calvin cycle enzymes such as glyceraldehyde-3-phosphate dehydrogenase (GAPDH), phosphoribulokinase (PRK), transketolase (TK), and small subunits of ribulose biphosphate carboxylase/oxygenase (RuBisCO) are highly expressed during phase I and then decline during phase III of berry development (Waters et al., 2005; Deluc et al., 2007). Analysis of gene expression in detached skins (Grimplet et al., 2007; Ghan et al., 2017) reveals a higher proportion of transcripts encoding proteins with functions related to photosynthesis and carbon assimilation compared to pulp (Grimplet et al., 2007), but this proportion decreases in late-ripening berries (Ghan et al., 2017).

Compared to gene expression studies, literature addressing photosynthesis in grape berries through proteomic approaches is much less abundant. In the skin of Cabernet Sauvignon berries, proteins involved in photosynthesis and carbohydrate metabolism were found overly accumulated at the beginning of color change, while those involved in energy and general metabolism decreased from the onset of ripening to the end of color change (Deytieux et al., 2007). Interestingly, pivotal proteins involved in photosynthesis were detected in the skin in higher amounts than in the pulp of ripe berries of Cabernet Sauvignon (Grimplet et al., 2009), including several light-harvesting components (chlorophyll *a*/*b*-binding proteins, photosystem II (PSII) components, oxygen-evolving enhancer protein 1) and enzymes involved in carbon fixation like the large subunit of RuBisCO.

In the present study, we wanted to clarify whether chloroplasts are kept in the anthocyanin-rich skin of mature red grape berries, where they may contribute to fruit metabolism by providing organic carbon, energy, or reducing power, as well as act as precursors for ripening-related pathways that start in that organelle. To address this hypothesis, plastids from green and mature grape exocarp were purified from cv. Vinhão (an important Portuguese red variety) for proteomic analysis. From a total of 4,852 proteins identified in the skin of chloroplastidial fractions, 1,053 entries were assigned to the chloroplast by bioinformatic tools, and 268 chloroplastic proteins were differentially accumulated between the green and mature phases. The results revealed that several proteins of the light reactions significantly accumulated in the skin chloroplasts at the mature stage, in parallel with a strong decrease in proteins involved in the reduction of NADP^+^ to NADPH and in the biosynthetic reactions of the Calvin cycle.

## Materials and methods

### Plant material

**Figure 1:**
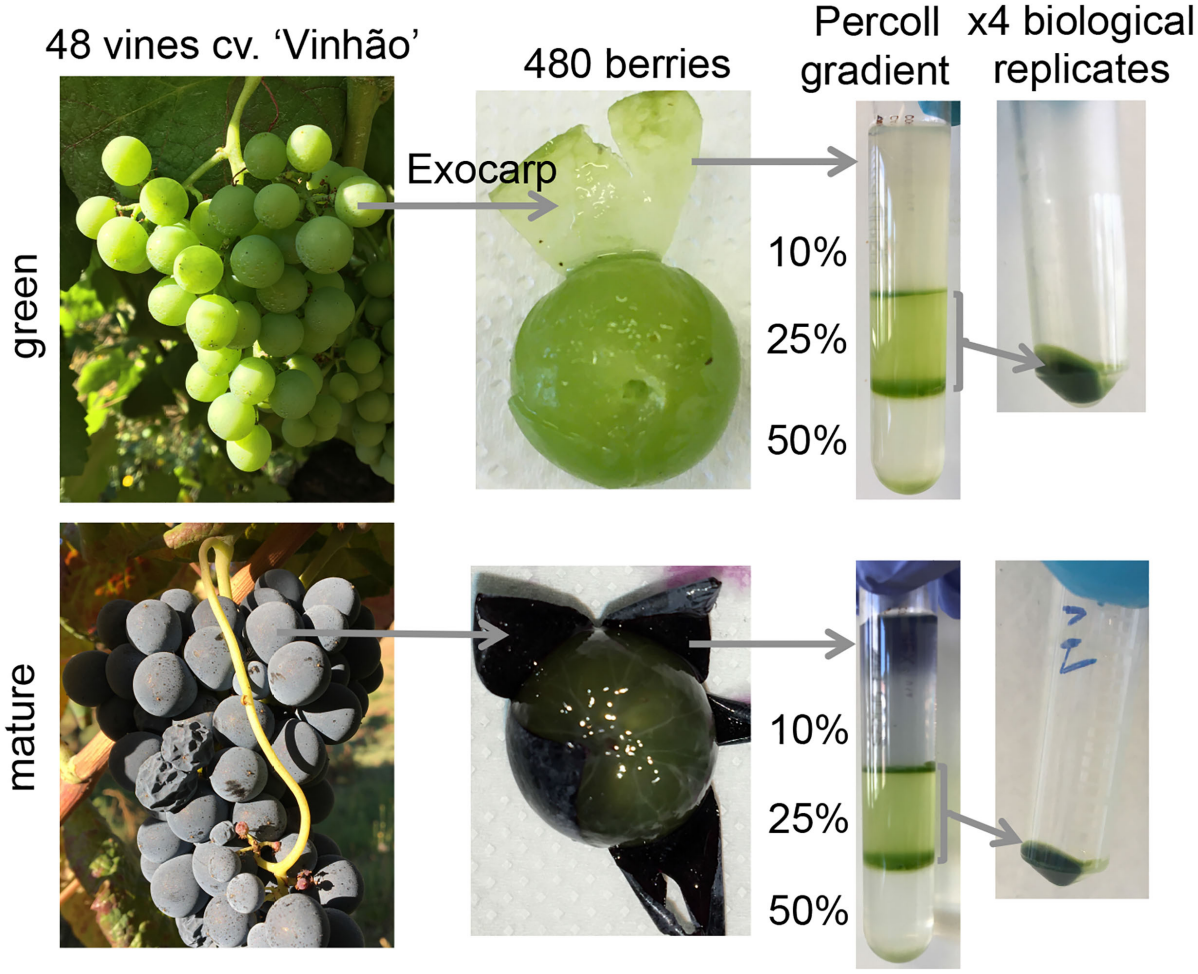
Plastid purification from the exocarp of Vitis vinifera cv. Vinhão at green (E-L 34) and mature (E-L 38) developmental stages.

Grape berries from the red cv. ‘Vinhão’ were collected in 2019 in a Portuguese ampelographic collection (Estaçaço Vitivinícola Amândio Galhano, N41°48′55.55′′/W8°24′38.07′′), located in the controlled appellation (DOC - Denominação de Origem Controlada) region of Vinhos Verdes in the northwest region of Portugal. Thirty-three-year-old vines were trained and spur-pruned on an ascendant simple cordon system. The soil was Cambic Umbrisol and acidic with low levels of phosphorous and potassium, rich in nitrogen, with low mineral colloids and high fertility at the first layer. Grape berries were sampled over four consecutive days at the late green (E-L 34) and mature (E-L 38) stages (Coombe, 1995) from 48 grapevines (12 grapevines per biological replicate) and transported to the laboratory in cooled containers. The exocarp of the berries was carefully separated from the mesocarp and used for plastid purification (**Figure 1**). From each vine, the seventh leaf from the apex was collected to ensure that measurements conducted on different dates correspond to leaves at a similar development stage.

### Plastid purification from the skin of grape berries

Exocarp tissues from 120 berries per replicate were homogenized in 200 ml of ice-cold extraction buffer (150 mM of Tris-HC1, pH 8.2, 5 mM of MgCl_2_, 5 mM of Ethylenediaminetetraacetic acid (EDTA), 500 mM of sorbitol, 2 mM of DL-Dithiothreitol (DTT), 0.5% of bovine serum albumin, and 2 mm of Phenylmethylsulfonyl fluoride (PMSF) in a Waring Blender (type 7012G) Waring Commercial Laboratory Blender (Waring Laboratories, Torrington, Conn., U.S.A. 2 × 7 s, maximum speed) (**Figure 1**). The homogenate was filtered through two layers of a 100-µm nylon mesh, transferred to 50-ml Falcon tubes, and centrifuged at 1,620×*g* for 10 min at 4°C (Eppendorf 5804R). The resulting crude organelle fraction was resuspended in 20 ml of the purification buffer (50 mM of Tris-HC1, pH 8.0, 1 mM of MgCl_2_, 1 mM of EDTA, 330 mM of sorbitol, 2 mM of DTT, 0.1% of Bovine Serum Albumin (BSA), and 2 mM of PMSF) and centrifuged at 150×*g* for 5 min at 4°C. The supernatant was recovered and centrifuged at 3,000×*g* for 10 min at 4°C; the pellet was resuspended in 5 ml of the purification buffer and layered to the top of 12 ml of discontinuous Percoll gradients (4 ml of 50%, 4 ml of 25%, 4 ml of 10% Percoll, dissolved in a purification buffer without BSA). The gradients were centrifuged at 4,500×*g* at 4°C for 10 min (A-4-44 rotor, Eppendorf 5804R centrifuge with medium acceleration and low deacceleration). The two middle bands (50%–25% and 25%–10% interfaces) that contained intact plastids were collected and washed twice in a purification buffer without BSA and centrifuged at 4,500×*g* at 4°C for 5 min to remove the Percoll gradients. The plastid pellet was finally resuspended in 1 ml of the purification buffer lacking BSA and centrifuged at 4,500×*g* at 4°C for 10 min, and the sediment was flash frozen in liquid nitrogen for proteomic analysis and chlorophyll quantification.

### Chlorophyll quantification

Chlorophyll quantification was performed accordingly with minor modifications (Lichtenthaler and Wellburn, 1983). Briefly, the above-described frozen pellets of the plastidial fraction were freeze-dried (Christ Alpha 2-4 LD Plus lyophilizer) and dissolved in 0.4 ml of acetone. Grapevine leaves and green berry skin were pulverized in liquid N_2_, and ±50 mg of each tissue was extracted in 1 ml of acetone. The samples were centrifuged at 14,000×*g*, and the absorbance of the supernatants was measured at 661 and 644 nm. Chlorophylls *a* and *b* and *a* + *b* were quantified using the following equations: C*a* = 11.24A_661_ – 2.04A_644_; *Cb* = 20.13A_644_ – 4.19A_661_, C*a* + *b* = 7.05A_644_ + 18.09A_644_, where the different values correspond to the absorption coefficients of acetone-specific pigments and A corresponds to the absorbance obtained in each wavelength. Values were normalized by the total protein amount that was determined spectrophotometrically by Bradford assay (Bradford, 1976).

### Sample preparation for proteomic analysis

Proteins were extracted from purified plastid preparations according to the procedure described by Tamburino et al. (2017). In brief, isolated plastid pellets were resuspended in a buffer containing 100 mM of Tris-HCl (pH 7.5), 100 mM of EDTA, 50 mM of Borax, 50 mM of ascorbic acid, 2% (w/v) of 2-mercaptoethanol, 30% (w/v) of sucrose, and 1 mM of PMSF. Samples were vortexed for 5 min, and then an equal volume of Tris-saturated phenol (pH 8.0) was added before vortexing again for 10 min. Samples were centrifuged at 15,000×*g* for 15 min at 4°C, and the upper phenolic phase was transferred to a new tube. Five Volumes of ammonium sulfate-saturated methanol were added to precipitate proteins, and samples were incubated at -20°C for 48 h. Samples were centrifuged at 15,000×*g* for 30 min at 4°C, and the protein pellets were washed once in methanol (ice-cold) and three times with acetone (ice-cold). In each washing step, protein pellets were centrifuged at 15,000×*g* for 15 min at 4°C. The final pellets were air-dried at room temperature, resuspended in a buffer containing 0.1 M of Tris-HCl (pH 8.5) and 1% of Sodium dodecyl sulfate (SDS), and stored at -20°C. The protein concentration was determined in subsamples again resuspended in 0.1 M of Tris-HCl (pH 8.5) and 0.1% of SDS, using the Bradford reagent (Bradford, 1976) and BSA as the standard.

The samples (10 µg) were reduced with dithiothreitol (100 mM for 60 min at 37°C) and alkylated in the dark with iodoacetamide (5 µmol for 20 min at 25°C). The resulting protein extract was washed with 2 M of urea with 100 mM of Tris-HCl and then with 50 mM of ammonium bicarbonate for digestion with endoproteinase Lys-C (1:10 w:w at 37°C, o/n, Wako, cat # 129-02541) and then for trypsin digestion (1:10 w:w for 8 h at 37°C, Promega cat # V5113) following (Wiśniewski and Mann, 2009) using the FASP Protein Digestion Kit procedure. After digestion, the peptide mix was acidified with formic acid and desalted with a MicroSpin C18 column (The Nest Group,Inc. Ipswich, USA) prior to LC-MS/MS analysis.

### Chromatographic and mass spectrometric analysis

Samples were analyzed using an Orbitrap Eclipse mass spectrometer (Thermo Fisher Scientific, San Jose, CA, USA) coupled with an EASY-nLC 1200 (Thermo Fisher Scientific, Proxeon, Odense, Denmark). Peptides were loaded directly onto the analytical column and were separated by reversed-phase chromatography using a 50-cm column with an inner diameter of 75 μm and a spectrometer packed with 2 μm of C18 particles (Thermo Scientific, San Jose, CA, USA).

Chromatographic gradients started at 95% of buffer A and 5% of buffer B, with a flow rate of 300 nl/min for 5 min, and gradually increased to 25% of buffer B and 75% of buffer A in 79 min and then to 40% of buffer B and 60% of buffer A in 11 min. After each analysis, the column was washed for 10 min with 10% of buffer A and 90% of buffer B. Buffer A was 0.1% formic acid in water. Buffer B was 0.1% formic acid in 80% acetonitrile.

The mass spectrometer was operated in a positive ionization mode with the nanospray voltage set at 2.4 kV and the source temperature at 305°C. The acquisition was performed in data-dependent acquisition (DDA) mode. Full MS scans with one micro scan at a resolution of 120,000 were used over a mass range of 350–1,400 m/z in the Orbitrap mass analyzer. Auto gain control (AGC) was set to ‘auto’ and charge state filtering disqualifying singly charged peptides was activated. In each cycle of data-dependent acquisition analysis, following each survey scan, the most intense ions above a threshold ion count of 10,000 were selected for fragmentation. The number of selected precursor ions for fragmentation was determined by the “Top Speed” acquisition algorithm and a dynamic exclusion of 60 s. Fragment ion spectra were produced *via* high-energy collision dissociation (HCD) at a normalized collision energy of 28%, and they were acquired in the ion trap mass analyzer. AGC was set to 2E4, and an isolation window of 0.7 m/z and a maximum injection time of 12 ms were used.

Digested bovine serum albumin (New England Biolabs cat # P8108S) was analyzed between each sample to avoid sample carryover and to assure the stability of the instrument, and QCloud (Chiva et al., 2018) was used to control the instrument’s longitudinal performance during the project.

### Data analysis

Acquired spectra were analyzed using the Proteome Discoverer software suite (v. 2.4, Thermo Fisher Scientific) and the Mascot search engine (v. 2.6, Matrix Science) (Perkins et al., 1999). The data were sought against a *Vitis vinifera* database (Canaguier et al., 2017), a list of common contaminants, and all the corresponding decoy entries (Beer et al., 2017). For peptide identification, a precursor ion mass tolerance of 7 ppm was used for MS1 level, trypsin was chosen as the enzyme, and up to three missed cleavages were allowed. The fragment ion’s mass tolerance was set to 0.5 Da for the MS2 spectra. Oxidation of methionine and N-terminal protein acetylation were used as variable modifications whereas carbamidomethylation on cysteines was set as a fixed modification. The false discovery rate (FDR) in peptide identification was set to a maximum of 5%.

Peptide quantification data were retrieved from the precursor ion area detector node of the Proteome Discoverer (v. 2.4) using 2 ppm mass tolerance for the peptide extracted ion current (XIC). The values normalized by total peptide amount were used to calculate the protein fold change *p*-values and the adjusted *p*-values of mature berry vs. green berry (**Figure S1**).

### Subcellular localization of proteins from their amino acid sequences

From the normalized data set, proteins present in at least three of the four replicates for each condition were retrieved and filtered using the *Vitis vinifera* proteome database universe (Canaguier et al., 2017) with the Qiime tool (v. 1.9.1, from the Galaxy project) (Afgan et al., 2018). The retrieved sequences were analyzed with three online bioinformatic tools (DeepLoc 1.0, Predotar, and TargetP 2.0), to recognize sorting signals and predict the subcellular localization of proteins from their amino acid sequences (Small et al., 2004; Almagro Armenteros et al., 2017; Salvatore et al., 2019). Principal component analysis (PCA) was performed in R software (v. 4.1.0) using the mixOmics package (v. 6.16.3) (Rohart et al., 2017).

The differentially accumulated proteins (DAP) that had been sorted into plastid/chloroplast by the three tools were retrieved for further analysis.

### Gene Ontology term, Kyoto Encyclopedia of Genes and Genomes pathway enrichment, MapMan analysis, and protein interaction network

To perform a Gene Ontology (GO) term enrichment and KEGG (Kyoto Encyclopedia of Genes and Genomes) pathway enrichment analysis on the predicted chloroplastidial DAPs, the *Vitis vinifera* ENSEMBL annotations of DAPs were converted to ENTREZID *via* the “bitr” function from ClusterProfiler package (v. 4.0) (Yu et al., 2012). The ID was used as a reference for the search of the GO terms. To match the ID to the GO terms in a fast and reliable way, an SQLite annotation data package was created using a modified version of the popular AnnotationForge R package, adapted to work with plant genomes (Carlson and Pagès, 2019), according to the R script published by (Santos et al., 2020).

The biological process (BP), molecular function (MF), and cellular component (CC) of the GO-term enrichment analysis of chloroplastidial proteins were performed separately for both the less and more accumulated proteins with the “enrichGO” function from the R package clusterProfiler, with the DAPs as the universe, Benjamini-Hochberg for the pAdjustMethod, pvalueCutoff = 0.05, qvalueCutoff = 0.05, and the above-described annotation-created data package as OrgDb. Due to the hierarchical nature of gene ontologies, a semantic reduction of GO terms using the “rrvgo” R package (v. 1.4.0) was performed, grouping similar terms based on their semantic similarity. For both the less and more accumulated proteins, the similarity matrices of the biological process, molecular function, and cellular component’s GO terms were created using the “Rel” (Relevance) as the method and “org.At.tair.db” (*Arabidopsis thaliana*) as a reference database. Similarity matrices were reduced using a threshold of 0.7. KEGG pathway enrichment was performed with the “enrichKEGG” function from the clusterProfiler, using the same conditions of GO enrichment with *Vitis vinifera* (vvi) as an organism.

The chloroplastidial DAPs were categorized with MapMan standalone software (v. 3.5.1) (Thimm et al., 2004), and the results were visualized in MapMan pathways.

To construct a protein–protein interaction (PPI) network for a protein module, we used the open-source database of known and predicted protein interactions STRING (v. 11.5, http://string-db.org) (Szklarczyk et al., 2019). The function protein query was used under the criteria for linkage with experiments, co-expression, databases, experiments, and textmining, with the default settings (medium confidence score: 0.400, network depth: 0 interactions). The resulting network and protein description files were used to produce the networks in Cytoscape 3.8.2 (http://www.cytoscape.org). Additionally, we used clusterMaker2 (v. 1.2.1) to perform Markov clustering (MCL) (Enright et al., 2002) of the protein network to obtain the subnetworks.

## Results

### Chloroplasts and chlorophylls in the skin of green and mature berries

**Figure 2:**
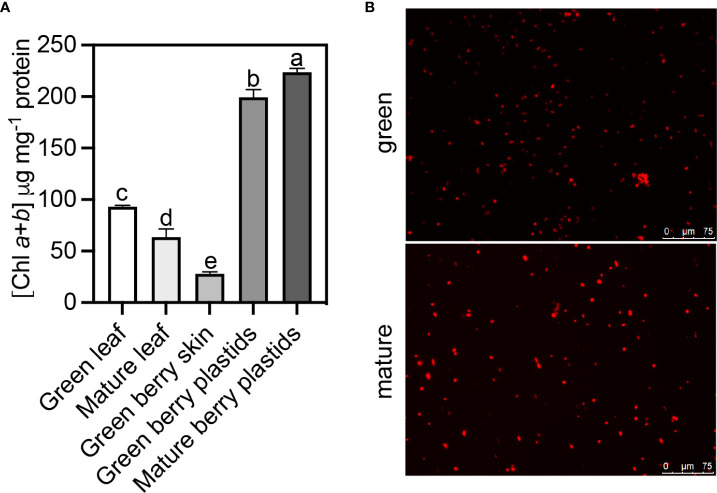
Plastid purification from the exocarp of Vitis vinifera cv. Vinhão at green (E-L 34) and mature (E-L 38) developmental stages. **(A)** Total chlorophyll content (a + b) present in grapevine leaves at the green and mature stages, in berry skin at the green stage, and in the purified plastids of grape berry skin at the green and mature stages of development and **(B)** visualized by fluorescence microscopy. One-way ANOVA with Tukey’s post-hoc test; different letters denote statistical differences between bars.

The chlorophyll content in the skin tissues of green berries was three- and two-fold lower than in leaves sampled at the green and mature stages of berry development, respectively (**Figure 2A**). Purified plastids from the skin tissues of green and mature berries were observed under a fluorescence microscope (**Figure 2B**), and the corresponding chlorophyll content is depicted in **Figure 2A**. Unlike the tomato fruit that undergoes a physiological transition during ripening on the differentiation of photosynthetically active chloroplasts into chromoplasts, **Figure 2** shows that in the mature berries of red grapes, the transition in color is not associated with a loss of chloroplasts in the skin, and the plastid content in total chlorophylls also did not decrease (**Figure 2A**). In green berries, the amount of total chlorophyll per mg^–1^ of protein increased from 28 in skin tissues to 200 in purified plastids, which shows a good degree of chloroplast enrichment during purification.

### Gene Ontology annotation of differentially accumulated proteins identified in the skin chloroplasts

A total of 4,852 proteins present in at least three of four replicates were identified in the chloroplastidial fractions from the skins of both green and mature berries (**Supplementary Table 1**). The prediction of their subcellular localization was analyzed with three different algorithm tools. These tools revealed that a large part of the identified proteins localizes unequivocally in the chloroplast (**Supplementary Figure 2**). The concatenated results from the three sub-cellular bioinformatic tools showed 1,053 entries of chloroplastidial proteins (**Supplementary Figure 2A–C**; **Supplementary Table 2**), of which 268 were differentially accumulated (|log2FC| > 1.0; adjusted *p*-value ≤ 0.05) in the skin of both green and mature berries (**Supplementary Table 3**). The principal component analysis of the differentially accumulated chloroplastidial proteins showed a clear separation indicating that the proteome of this organelle is distinct between the green and mature developmental stages (**Supplementary Figure 2D**).

**Figure 3:**
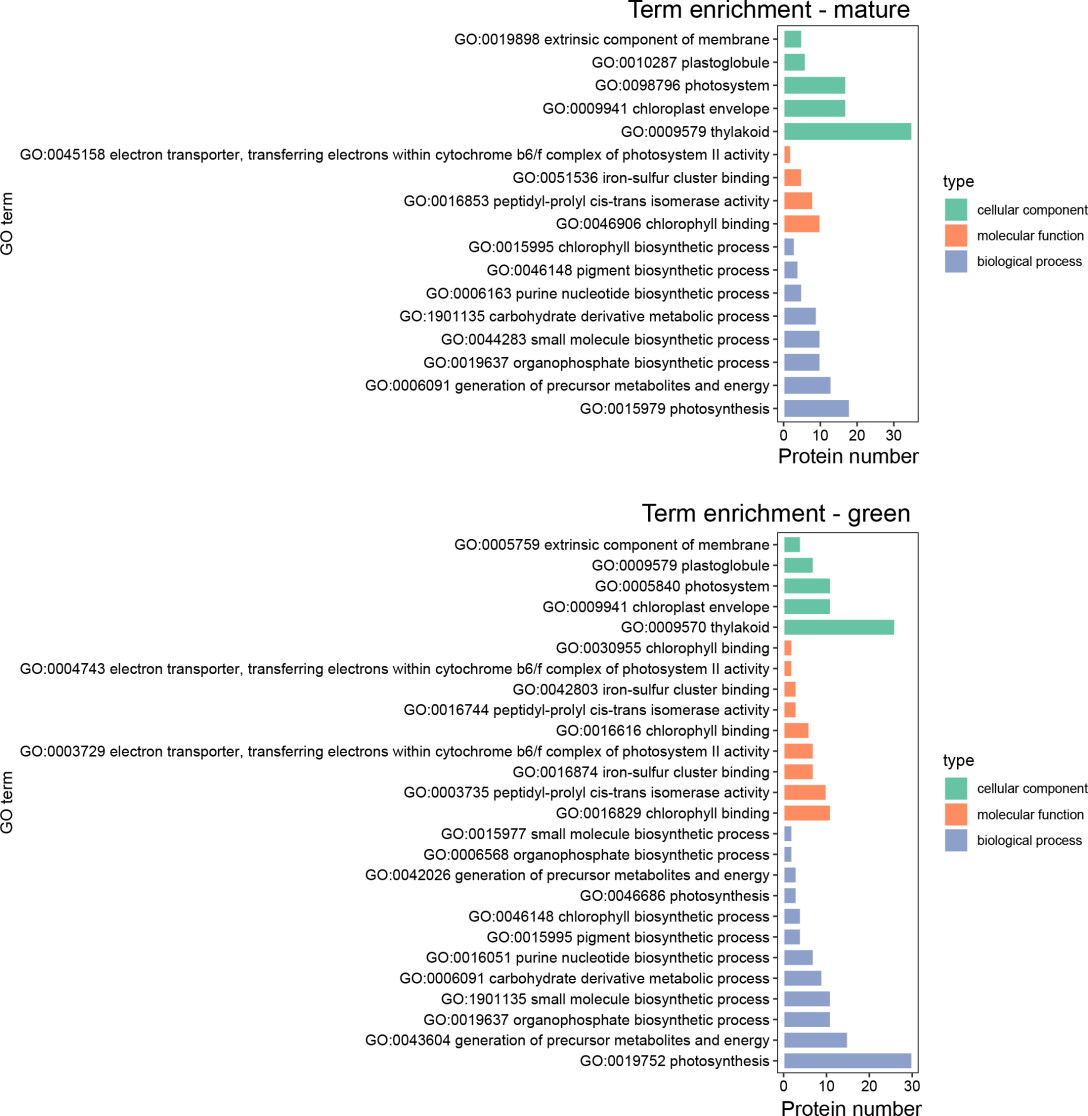
Gene Ontology (GO) term enrichment of differentially accumulated proteins (DAPs) in the chloroplasts of Vitis vinifera cv. Vinhão exocarp at the mature stage (E-L 38) vs. the green stage (E-L 34).

The Gene Ontology enrichment analysis of chloroplastidial differentially accumulated proteins was conducted to assess which biological processes of the three sub-ontologies (biological process, BP; molecular function, MF; and cellular component, CC) incurred significant changes in the transition between the green and mature stages (**Figure 3**). The GO terms enriched in chloroplastidial proteins from the skins of green berries (negative LogFC values) were the BP sub-ontology for photosynthesis, with the highest number of enriched terms, followed by the “generation of precursor metabolites and energy.” The MF sub-ontology also showed more enriched terms at the green stage. Regarding CC, chloroplastidial proteins revealed similar enriched GO terms at both developmental stages. In the chloroplasts from the skins of mature berries, the photosynthesis, generation of precursor metabolites and energy, and organophosphate biosynthetic process were the most enriched terms for the BP sub-ontology; chlorophyll-binding was the most enriched term for the MF sub-ontology; lastly, the thylakoid and chloroplast envelope or photosystem were the most enriched terms for the CC sub-ontology.

### Kyoto Encyclopedia of Genes and Genomes and MapMan annotation of differentially accumulated proteins identified in the skin chloroplasts

Five enriched KEGG pathways were assigned to proteins more accumulated in the skin chloroplasts at the mature stage, including photosynthesis and photosynthesis-antenna pathways. The proteins contributing to the enrichment of the photosynthesis pathway are involved in photosystem repair, synthesis of ATP, or electron transfer between the photosystems, while the proteins contributing to the enrichment of the photosynthesis-antenna pathway are involved in light-harvesting and delivery of excitation energy to photosystems (**Supplementary Table 4**).

**Figure 4:**
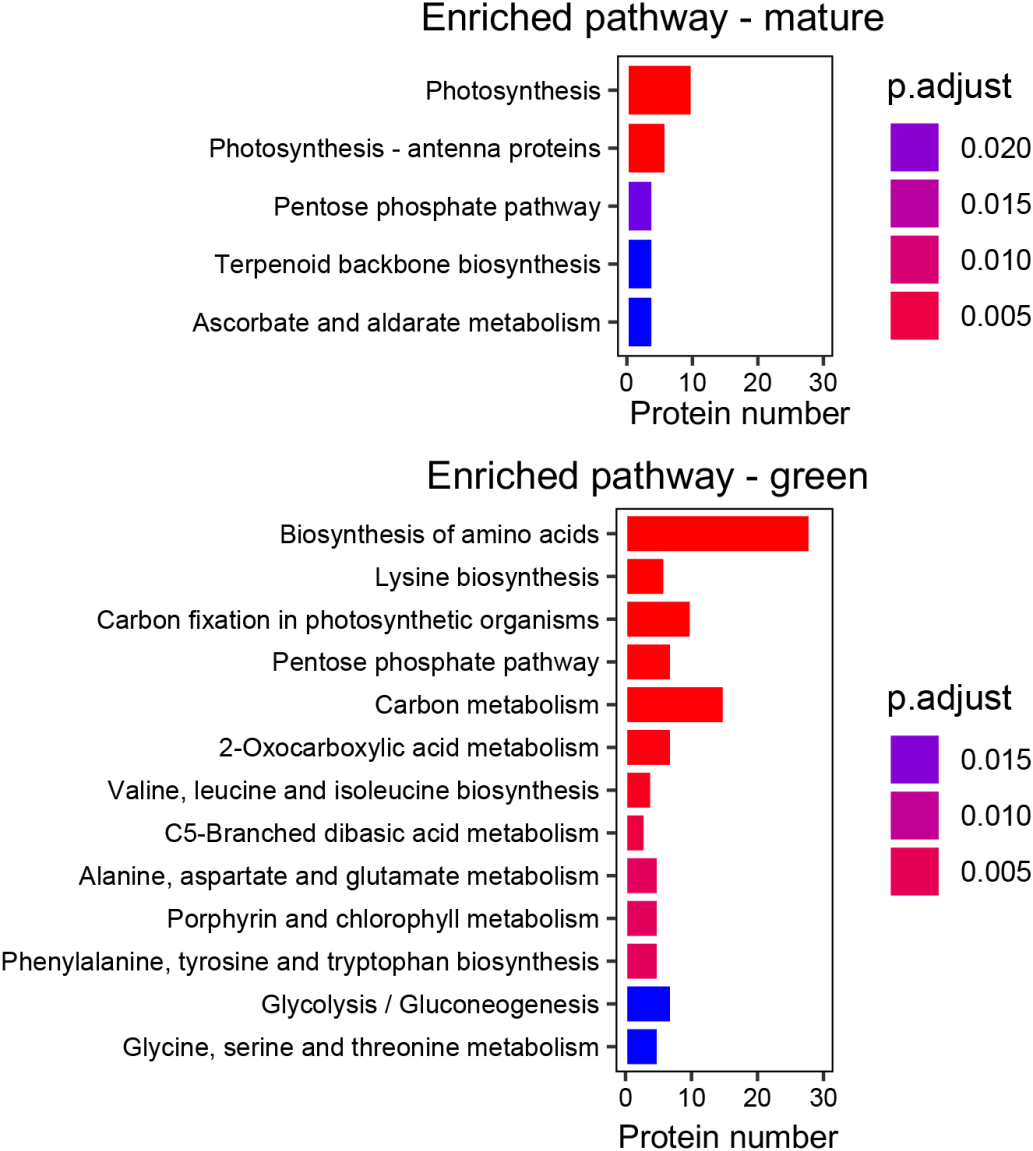
Kyoto Encyclopedia of Genes and Genomes (KEGG) pathway enrichment of differentially accumulated proteins (DAPs) in the chloroplasts of Vitis vinifera cv. Vinhão exocarp at the mature stage (E-L 38) vs. the green stage (E-L 34).

In the skin chloroplasts at the green stage, the biosynthesis of amino acids was the most enriched pathway (28 proteins) with proteins such as ribulose-phosphate 3-epimerase, involved in the initial steps of amino acid biosynthesis, or chorismate mutase and tryptophan synthase, involved in the biosynthesis of the aromatic amino acids phenylalanine, tyrosine, and tryptophan. Only two of the seven DAPs found as involved in the aromatic amino acid biosynthetic pathway were more accumulated at the mature stage. The carbon metabolism biosynthetic pathway was the second most-enriched pathway in the green stage with proteins such as malate dehydrogenase, involved in the tricarboxylic acid biosynthesis; GAPDH, involved in the glycolysis; or transaldolase, involved in the biosynthesis of pentose phosphate (**Figure 4**).

**Figure 5:**
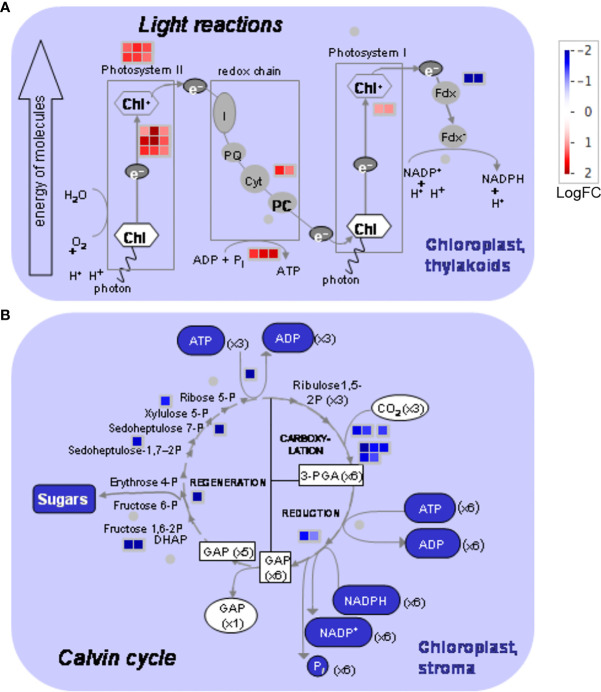
MapMan photosynthesis overview obtained from differentially accumulated proteins (DAPs) in the chloroplasts of Vitis vinifera cv. Vinhão exocarp at the mature stage (E-L 38) vs. the green stage (E-L 34). **(A)** Light reactions and **(B)** Calvin cycle. In red, proteins more accumulated in mature berries; in blue, proteins more accumulated in green berries. The MapMan figure was made by MapMan Version 3.6.0rc1 (https://mapman.gabipd.org/).

MapMan analysis (**Figure 5A**) showed that several proteins of the light reactions significantly accumulated in the skin chloroplasts at the mature stage (up to 11-fold): six proteins from the photosystem II light-harvesting complex (LHC) (bin 1.1.1.1); nine PSII polypeptide subunits (bin 1.1.1.2); five proteins from the redox chain, including two from cytochrome *b6/f* (bin 1.1.3); and two polypeptide subunit proteins of photosystem I (PSI). ATP synthase beta and delta subunits (bins 1.1.4.2 and 1.1.4.7) also accumulated at the mature stage, suggesting that energy production is stimulated. Conversely, two ferredoxin-NADP^+^ reductase proteins (bin 1.1.5.2) were less abundant (up to a 21-fold change decrease from the green to mature stage), suggesting that the production of NADPH that feeds the Calvin cycle is downregulated at this stage. Correspondingly, proteins involved in biosynthetic reactions in the chloroplast, such as ribulose phosphate carboxylases (bin 1.3.1), RuBisCO-interacting proteins (bins 1.2.13 and 1.3.2), glyceraldehyde-3-phosphate dehydrogenases (bin 1.3.4), fructose-biphosphate aldolases (bin. 1.3.6), sedoheptulase-1,7-biphosphatase (bin 1.3.9), ribulose-5-phosphate-3-epimerase (bin 1.3.11), and PRK (bin 1.3.12) also decreased from the green to the mature stage up to 19-fold (**Figure 5B**). Remarkably, transketolase (bin 1.3.8), decreased 600-fold. Some chloroplastidial proteins like phosphoglycolate phosphatase (bin 1.2.1), glycolate oxidase (bin 1.2.2), glycine cleavage (1.2.4.2/.4), and hydroxypyruvate reductase (bin 1.2.6) (**Supplementary Table 5**) related to photorespiration also accumulated more at the green stage.

### Protein–protein interaction network of mapped chloroplastidial differentially expressed proteins

**Figure 6:**
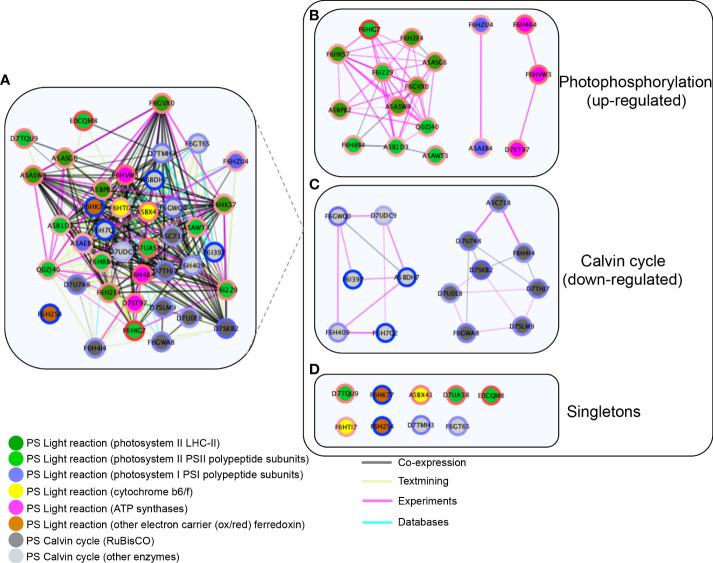
Network protein–protein interactions of differentially accumulated proteins (DAPs) in the chloroplasts of Vitis vinifera cv. Vinhão exocarp at the mature stage (E-L 38) vs. the green stage (E-L 34). **(A)** Functional association cluster network. Network limited to “physical interaction” clusters. **(B)** Photophosphorylation-involved proteins; **(C)** Calvin cycle involved proteins, and **(D)** Singletons. LogFC ratios of protein abundance between the green and mature stages were mapped around the nodes using a blue (negative) and red (positive) color gradient. The inner nodes were colored according to the MapMan bins. The edge scores are visualized by co-expression (black), co-occurrence (blue), experiments (pink), and textmining (light green). The network clusters were obtained using Markov clustering on the STRING network of proteins from panel **(A)**. Proteins were named according to their canonical name.

String network analysis was performed to reveal the PPI network of 40 significant DAPs assigned to the chloroplast (**Figure 6A**, **Supplementary Table 6**). The PPI network (default score filter of 0.4) of 40 nodes and 374 edges revealed significantly more interactions than expected (expected number of edges = 21, *p*-value <1.0e-^16^), indicating that the proteins are at least partially biologically connected as a group. The network showed a functional association tight cluster of co-expressed proteins assigned to light reaction photosystems and the Calvin cycle (**Figure 6A**). The proteins highly accumulated at the mature stage (red circles) and were involved in light reaction photosystems (green nodes) co-expressed with the ones involved in the Calvin cycle reactions (gray nodes) and more accumulated at the green stage (blue circles). Several of these co-expression interactions were experimentally obtained (pink edges) (**Figure 6A**). The network subset limited to “physical interaction” revealed three modules where proteins with the same expression pattern correlated (**Figure 6B**). Photosystem II LHC-II proteins correlated with PSII polypeptide subunits, while PSI polypeptide subunits correlated with the light reaction ATP synthases. The proteins involved in the Calvin cycle were classified into two clusters: fructose biphosphate aldolases that correlated with GAPDH and PRK in one cluster (light gray nodes) and RuBisCO subunits that correlated with another cluster (dark gray nodes). These clusters reveal a strong physical interaction among proteins with similar expression patterns.

## Discussion

### The skin of the green and mature grape berries is rich in chloroplasts

A continuous decrease in chlorophyll content during ripening has been observed in different grape berries of red varieties (Giovanelli and Brenna, 2007; Kamffer et al., 2010). In whole cv. Merlot berries, the amount of chlorophyll pigments decreased from 19 to 10 μg/berry fresh weight, but in our study that targeted the skin of the red cv. Vinhão grapes, the number of chloroplasts and their relative content in chlorophylls remained high at the mature stage (**Figures 2A, B**). Conversely, a decrease in chlorophyll content was observed in the leaves of cv. Vinhão, in agreement with previous results (Bertamini and Nedunchezhian, 2002; Casanova-Gascón et al., 2018). The degradation of chlorophyll in leaves can result from a genetically programmed senescence process and in response to environmental factors such as temperature and low humidity (Casanova-Gascón et al., 2018). Our results thus strongly suggest that the skin of mature berries of cv. Vinhão is rich in chloroplasts and anthocyanins, which are known to accumulate after veraison. Supporting histological studies showed a clear colocalization of chlorophylls and anthocyanins in the skin of ripe berries of cv. Pinot Noir (Agati et al., 2007). Because anthocyanins protect the photosynthetic machinery in leaves (Gould, 2004; Lo Piccolo et al., 2018) and may retard leaf senescence (Lo Piccolo et al., 2018), one cannot discard that they may have similar protective roles in the berry skin of red cultivars. Hence, unlike tomatoes, where chlorophylls are replaced by carotenoids upon maturation, grape skins are likely to be kept photosynthetically competent until maturity. It is worth noting that chloroplast proteomes are different between the green and mature berries, further suggesting that photosynthesis in the skins of mature berries from cv. Vinhão may have relevant physiological roles, as discussed below (**Figures 3**, **4**).

### Light reactions are stimulated and the Calvin cycle is inhibited in the skin of mature grape berries

**Figure 7:**
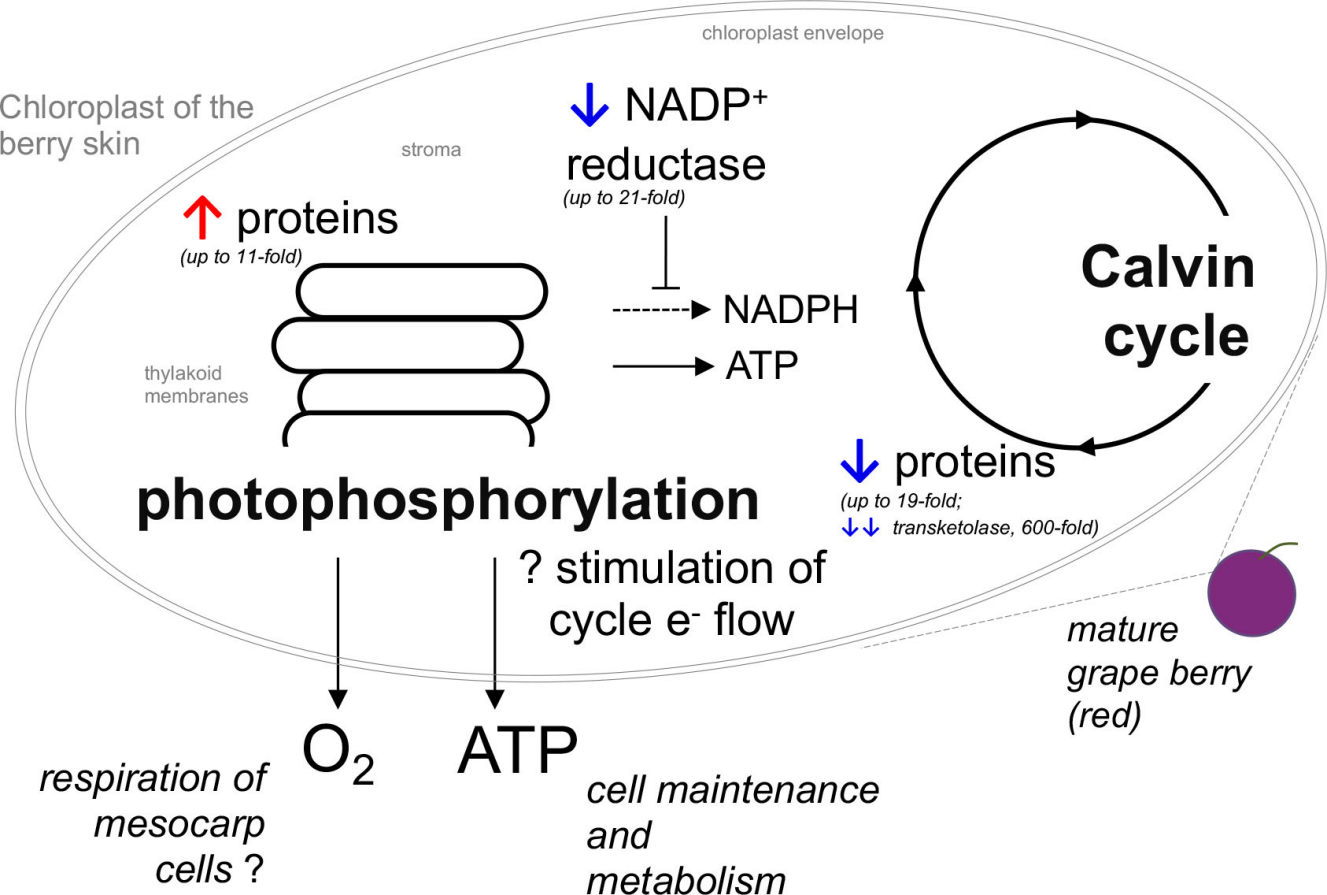
Illustration of the proposed changes in photosynthesis occurring in the chloroplasts of the skin of red grape berries during the transition from the green (E-L 34) to the mature stage (E-L 38). Blue and red arrows denote that the proteins are overrepresented (↑) or underrepresented (↓) at the mature stage when compared to the green stage.

The observation that several proteins of the light reactions were significantly accumulated in the skin chloroplasts at the mature stage, and that the transition from the green to mature stage was accompanied by a strong decrease in proteins involved in the biosynthetic reactions of the Calvin cycle, suggested that the skin of mature red berries has a major role in ATP production through the stimulation of cyclic electron flow. Among the 22 highly accumulated proteins in the skin of mature berries, we cite those from the antenna complex, PSII, PSI, cytochrome *b6/f*, and ATP synthase. It is worth noting that two ferredoxin-NADP^+^ reductase proteins were underrepresented up to 20.5-fold at the mature stage, suggesting that the reducing power of ferredoxin is being used to produce ATP through ATP synthase rather than NADPH for the Calvin cycle (**Figures 5**, **7**). The STRING network subset limited to ‘physical interaction’, evidenced well-defined modules were proteins with the same accumulation pattern, like (**Figure 6**). Conversely, among the 17 impoverished proteins from the Calvin cycle are those from RuBisCO and other key enzymes: ribulose-5-phosphate-3-epimerase, PRK, glyceraldehyde 3-phosphate, fructose 1,6-bisphosphate aldolase, sedoheptulose 1,7-bisphosphatase, and transketolase (600-fold). This strongly suggests that the synthesis of organic C compounds is switched off during the transition from the green to mature stage in the skin of the mature berries.

As reported in the Introduction, few studies have addressed the proteome of the grape berry as it relates to photosynthesis, energy, and C metabolism (Deytieux et al., 2007; Grimplet et al., 2009). To the best of our knowledge, we report here the first proteomics study on isolated chloroplast in the grape berry skin. In a proteomics approach conducted in detached skins of Cabernet Sauvignon, proteins involved in photosynthesis and carbohydrate metabolism, including RuBisCO and TK (found as impoverished at the mature stage in the present study), were identified as being overrepresented at the beginning of color change, while the end of color change was characterized by an overrepresentation of proteins involved in anthocyanin synthesis (Deytieux et al., 2007). In the present study, the proteins detected in the isolated chloroplasts of the skin, including chlorophyll *a*/*b* proteins, plastidic fructose-bis-phosphate aldolase (upregulated at the mature stage in the present study), and RuBisCO subunits or TK (found as impoverished at the mature stage in the present study), were also found to be more abundant in the skin than in the pulp of mature Cabernet Sauvignon berries (Grimplet et al., 2009), but their results are more difficult to compare with those of the present study. Specific changes in protein amounts throughout the berry formation and ripening processes were described in Muscat Hamburg (Martínez-Esteso et al., 2013), but that study was performed on berry mesocarp and thus, is not directly comparable to our study. Nonetheless, and in agreement with our results, a decrease in the abundance profiles up to the mature stage of several Calvin cycle proteins, including PRK and RuBisCO, was observed (in the mesocarp), with a sharper decrease observed from the green stage to the onset of veraison. This may suggest that the impoverishment of C-fixing proteins from the green to the mature stage occurs both in the pulp and in the skin of berries. However, contrary to the observations reported here, light reaction proteins, which displayed a moderate increase in abundance during the grape berry’s first growth period (EL-31 to EL-33), were impoverished during fruit ripening (Martínez-Esteso et al., 2013).

Previous transcriptomics approaches performed on berries of Corvina, Shiraz, Cabernet Sauvignon, Cabernet Franc, Merlot, Pinot Noir, Chardonnay, Sauvignon Blanc, and Semillon (in whole berries and skins) during development and ripening (Terrier et al., 2005; Waters et al., 2005; Deluc et al., 2007; Grimplet et al., 2007; Sweetman et al., 2012; Dal Santo et al., 2013; Ghan et al., 2017) showed that transcript-encoding proteins associated with photosynthesis-related functions, including photoreaction centers I (PCI) and II (PCII), chlorophyll *a*/*b*-binding proteins, photosystem II core complex proteins (enriched in the skin of mature berries in the present study) were strongly expressed during the phase I of berry development. On the other hand, genes encoding Calvin cycle enzymes such as glyceraldehyde-3-phosphate dehydrogenase, PRK, TK as well as RuBisCO small subunit (impoverished in the skin of mature berries in the present study) were highly expressed during phase I and then declined during phase III of berry development. In accordance with the present study, a strong decrease in proteins involved in biosynthetic reactions of the Calvin cycle was observed (**Figure 5**). Results from transcriptomic analysis in the detached skins of mature berries showed that this tissue is particularly rich in several light-harvesting and photosystem components, including cytochrome C6A and light-harvesting complex II type I, and that transcript abundance decreased in late ripening stages (Grimplet et al., 2007; Ghan et al., 2017).

Amino acid metabolism is tightly linked to energy and carbohydrate metabolism, protein synthesis, and secondary metabolism. In grapevines, the amino acids isoleucine, leucine, phenylalanine, and valine are precursors of higher alcohols and esters, which contribute to the desirable aromas in wines, while phenylalanine is the main substrate in the phenylpropanoid pathway that leads to the synthesis of important organoleptic compounds such as anthocyanins and stilbenes (Garde-Cerdán and Ancín-Azpilicueta, 2008). The present study showed that 28 proteins of the amino acid biosynthetic pathways were strongly impoverished from the green to the mature stage. A study on cv. Nebbiolo Lampia also showed a decrease in three enzymes of amino acid metabolism from the green to the mature stages (Giribaldi et al., 2007), while a proteome study on cv. Barbera grape skins showed an increase in the expression of seven proteins (10% of the functional categories) of the amino acid metabolism from veraison to the mature stage, from which five decreased until they reached full maturity (Negri et al., 2008).

The high respiratory demand of the berry tissues, particularly at the mature stage, entrains O_2_ deficiency that was recently reported to be associated with cell death in cv. Shiraz (Tilbrook and Tyerman, 2008; Xiao et al., 2019). This is particularly relevant in the context of ongoing climate warming because higher temperatures increase the respiratory rate of tissues. In this regard, the light reactions of chloroplasts on the skin are likely an important source of O_2_ for berry tissues when gas exchange is very limited at the mature stage, but they may also provide ATP and/or reduce power to other physiological processes, including anthocyanin synthesis, which is an energy-demanding process (**Figure 7**).

## Conclusions

Chloroplasts are key plant cell organelles where pivotal biochemical reactions occur, but their role in fruit ripening in several species is still far from being fully understood. For the first time, the present subcellular proteomic study focused on the analysis of chloroplastic proteins from the skin of green and mature grape berries. Our data confirmed that chloroplasts are present in mature berries but are masked by anthocyanins that accumulate during fruit maturation. Skin chloroplasts from green and mature berries have distinct proteomes that suggest different physiological roles; while proteins of the Calvin cycle are upregulated at the green stage, proteins involved in energy-yielding reactions predominate in mature red berries.

## Data availability statement

The mass spectrometry proteomics data have been deposited to the ProteomeXchange Consortium via the PRIDE partner repository with the dataset identifier PXD037346.

## Author contributions

AT: methodology, investigation, formal analysis, writing of the original draft, and review and editing of the draft. HN: conceptualization, methodology, investigation, formal analysis, and review and editing of the draft. MS: methodology, investigation, and review and editing of the draft. AF: conceptualization, methodology, review and editing of the draft. HG: conceptualization, resources, funding acquisition, writing of the original draft, and review and editing of the draft. All authors contributed to the article and approved the submitted version.

## Funding

This work was supported by the “Contratos-Programa” UIDB/04046/2020 and UIDB/ BIA/04050/2020 and by the core research project BerryPlastid (PTDC/BIA-FBT/28165/2017 and POCI-01-0145 - FEDER-028165). AT was supported by a postdoctoral researcher contract/position within the project BerryPlastid. HN was supported by an FCT postdoctoral grant (SFRH/BPD/115518/ 2016). AF was supported by UIDB/04046/2020 and UIDP/ 04046/2020 (to BioISI) Centre Grants from FCT, Portugal. MS was supported by FCT in the context of Norma Transitória — DL57/2016/CP[12345/2018]/CT[2475]. This work benefited from networking activities within the CoLAB Vines & Wines.

## Conflict of interest

The authors declare that the research was conducted in the absence of any commercial or financial relationships that could be construed as a potential conflict of interest.

## Publisher’s note

All claims expressed in this article are solely those of the authors and do not necessarily represent those of their affiliated organizations, or those of the publisher, the editors and the reviewers. Any product that may be evaluated in this article, or claim that may be made by its manufacturer, is not guaranteed or endorsed by the publisher.
