# Supplementary material for: A proteomic analysis shows the stimulation of light reactions and inhibition of the Calvin cycle in the skin chloroplasts of ripe red grape berries
Source: Front Plant Sci. 2022 Oct 25;13:1014532. doi: 10.3389/fpls.2022.1014532 (PMC9641181; doi:10.3389/fpls.2022.1014532)
Supplement: Supplementary file 2 [file Image_1.pdf]

A proteomic analysis shows stimulation of light reactions and inhibition of Calvin cycle in skin chloroplasts of ripe red grape berries

António Teixeira [a*], Henrique Noronha [a*], Mónica Sebastiana [b], Ana Margarida Fortes [b], Hernâni Gerós [a]

[a] Centre of Molecular and Environmental Biology (CBMA), Department of Biology, 2 University of Minho, Braga 4710-057, Portugal
[b] BioISI – Instituto de Biosistemas e Ciências Integrativas, Faculdade de Ciências, Universidade de Lisboa, 1749-016 Lisboa, Portugal

*Corresponding authors: antonio.teixeira@bio.uminho.pt; henriquenoronha@bio.uminho.pt

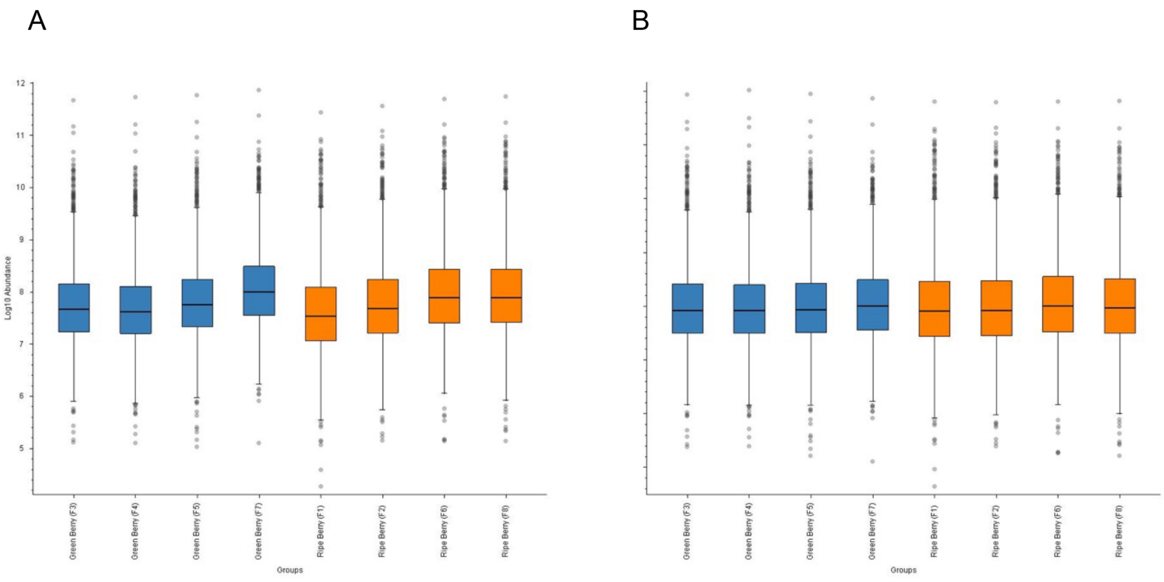

Figure S1. Total identified proteins from purified plastids from exocarp of *Vitis vinifera* cv 'Vinhão' at mature stage (E-L 38) *versus* green stage (E-L 34). (A) Abundance before normalization (B) Abundance after normalization.

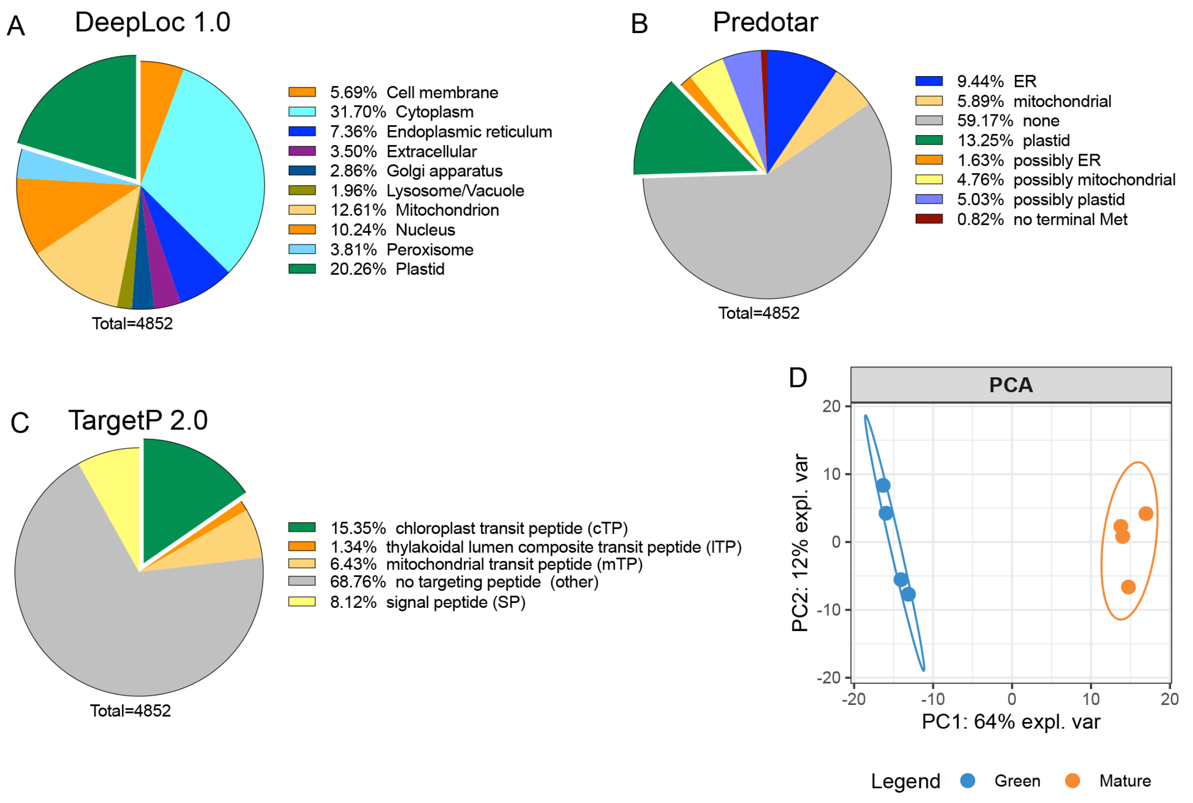

Figure S2. Protein subcellular localization prediction of total identified proteins from purified plastids of exocarp of *Vitis vinifera* cv 'Vinhão' at mature stage (E-L 38) *versus* green stage (E-L 34) by (**A**) DeepLoc 1.0, (**B**) Predotar and (**C**) TargetP 2.0. (**D**) Principal Component Analysis of proteins targeted to plastid.
